# Supplementary figures and images for: A class of deep intronic IGHMBP2 variants activate a shared cryptic splice donor, enabling correction of select variants with a single antisense oligonucleotide
Source: medRxiv. 2026 Apr 29:2026.04.20.26351111. Preprint. [Version 1] doi: 10.64898/2026.04.20.26351111 (PMC13317647; doi:10.64898/2026.04.20.26351111)

**Supplemental Figures**


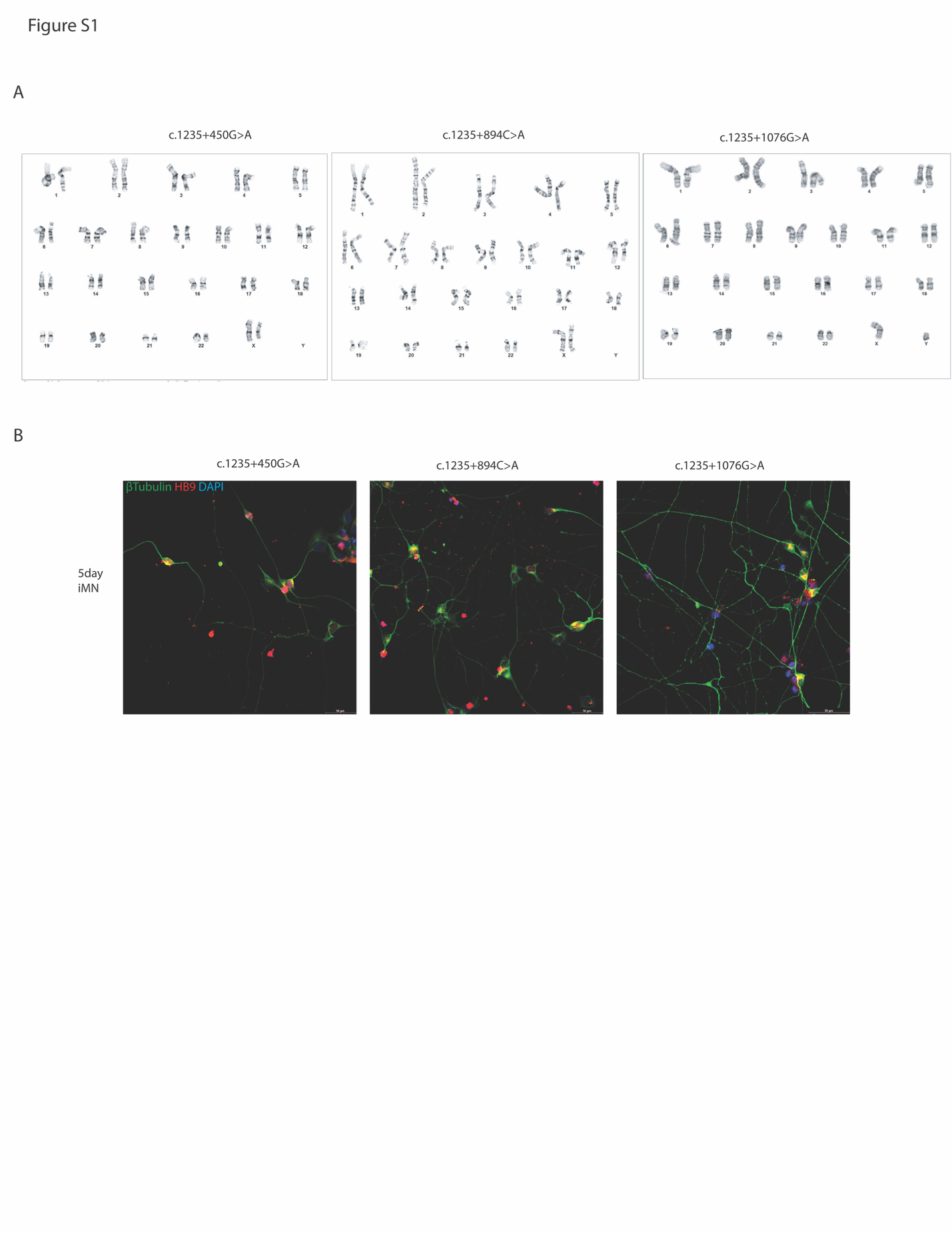





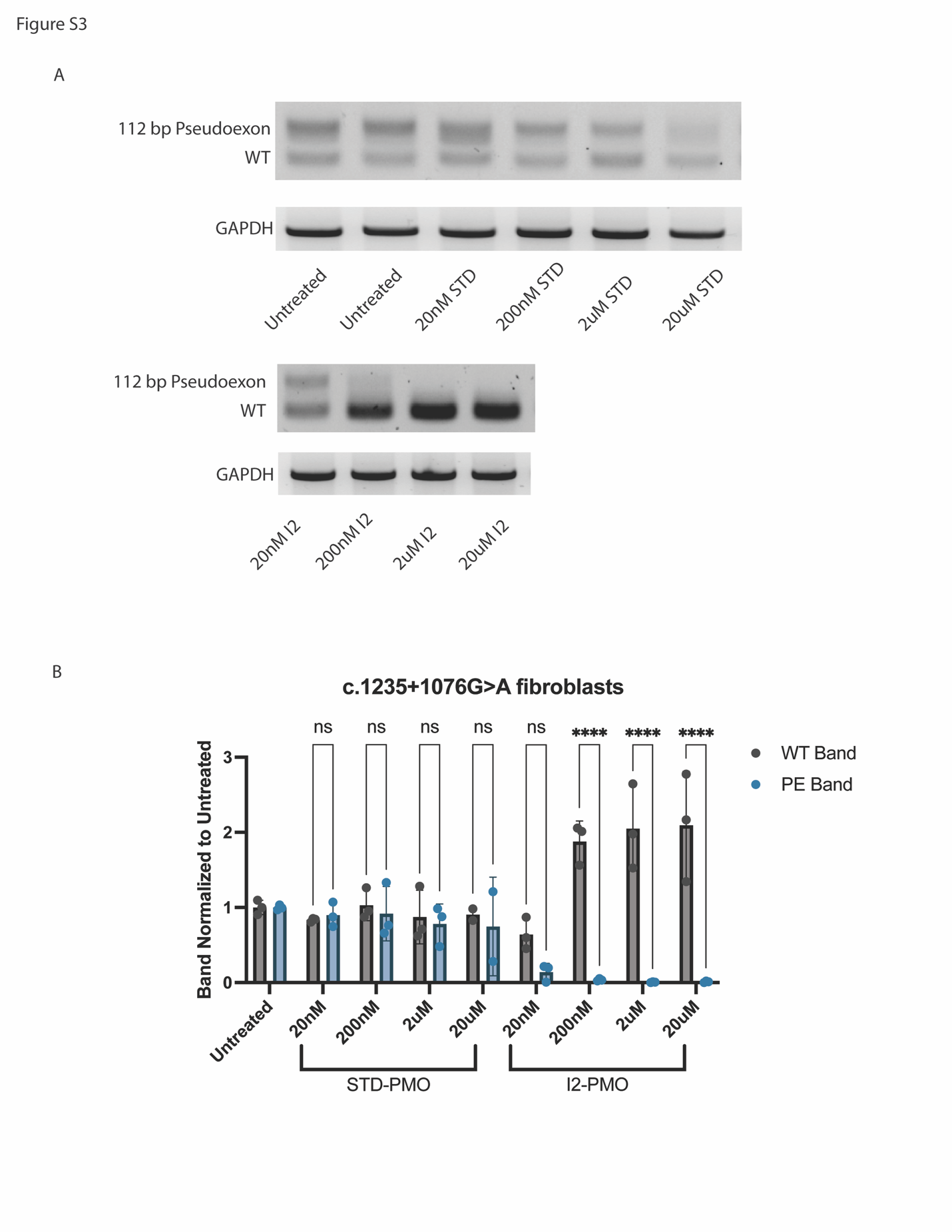








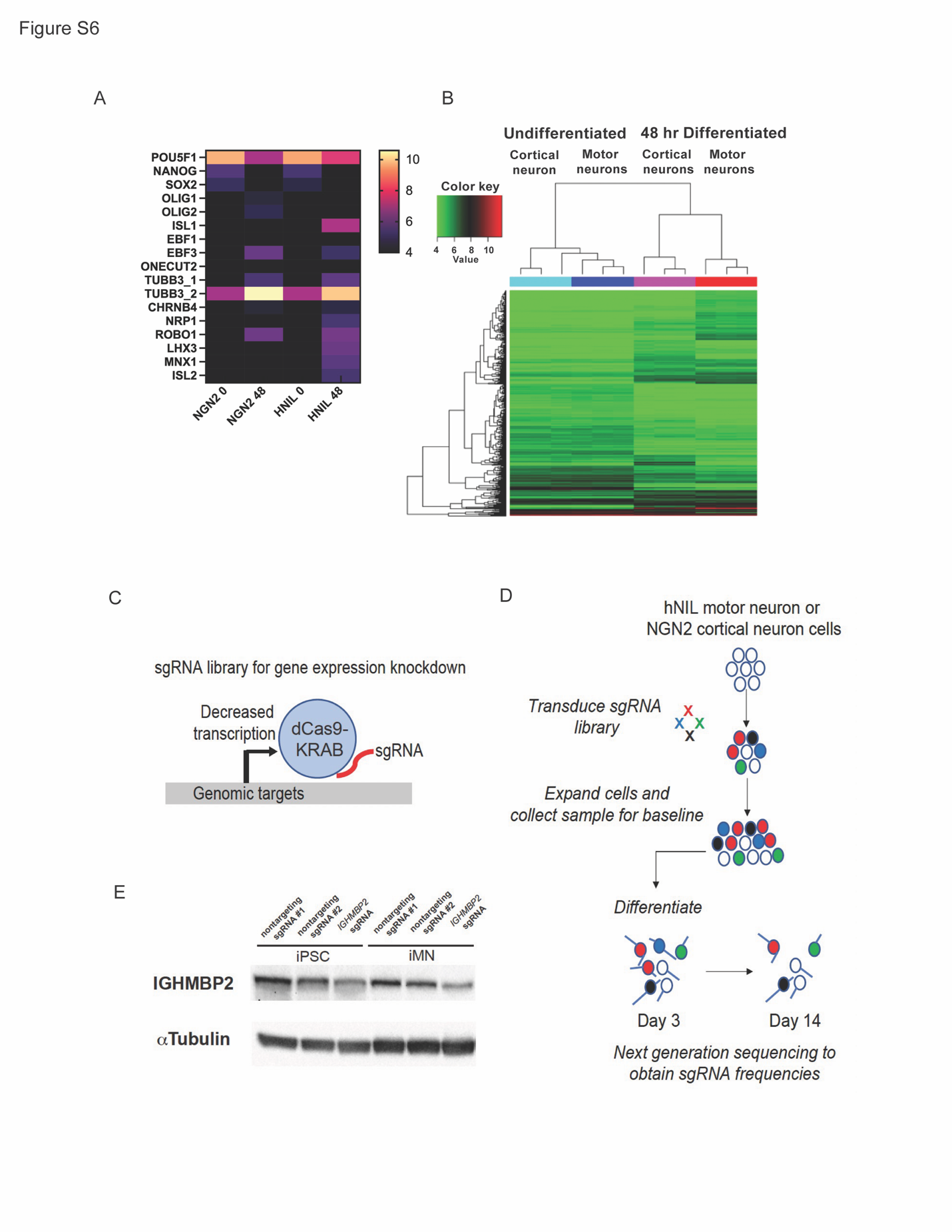














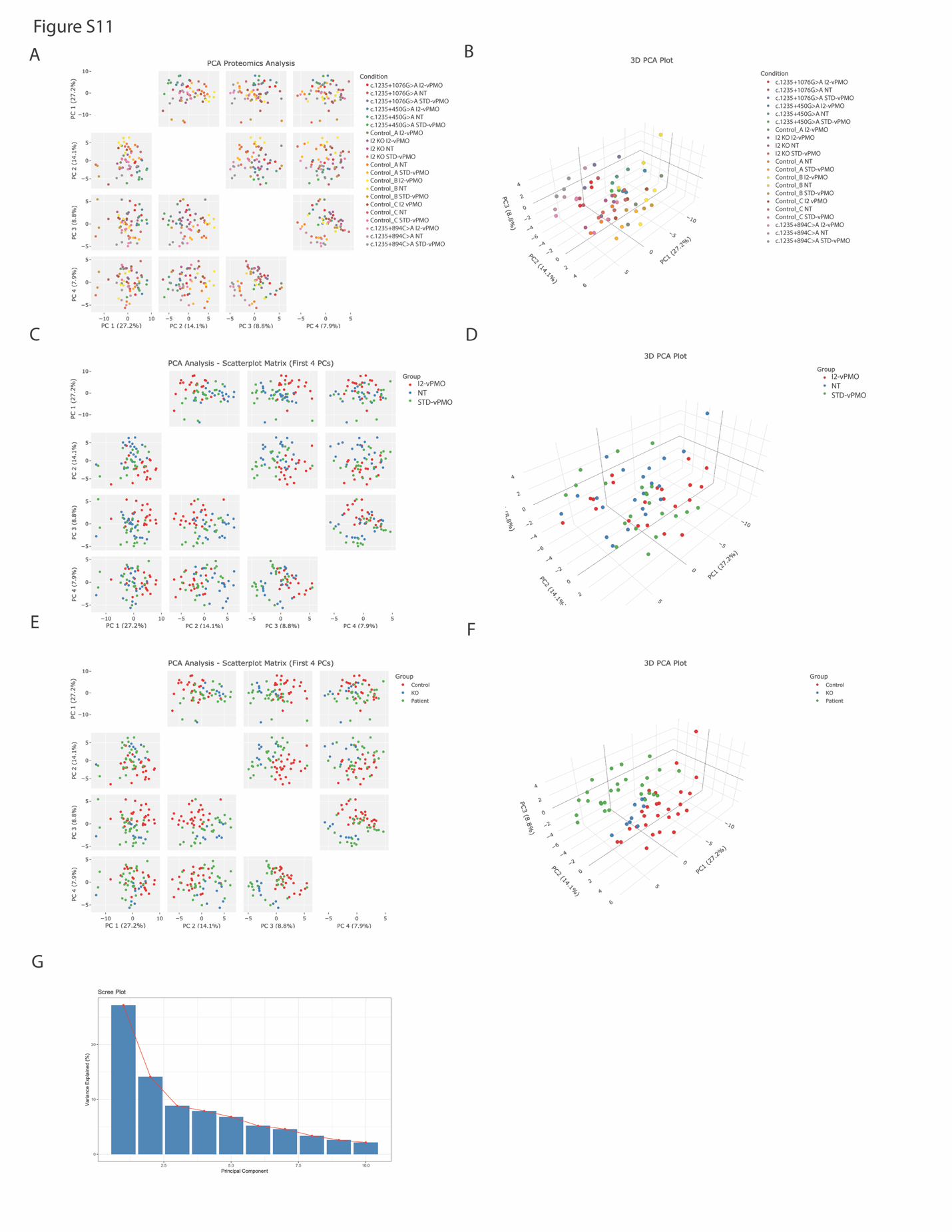








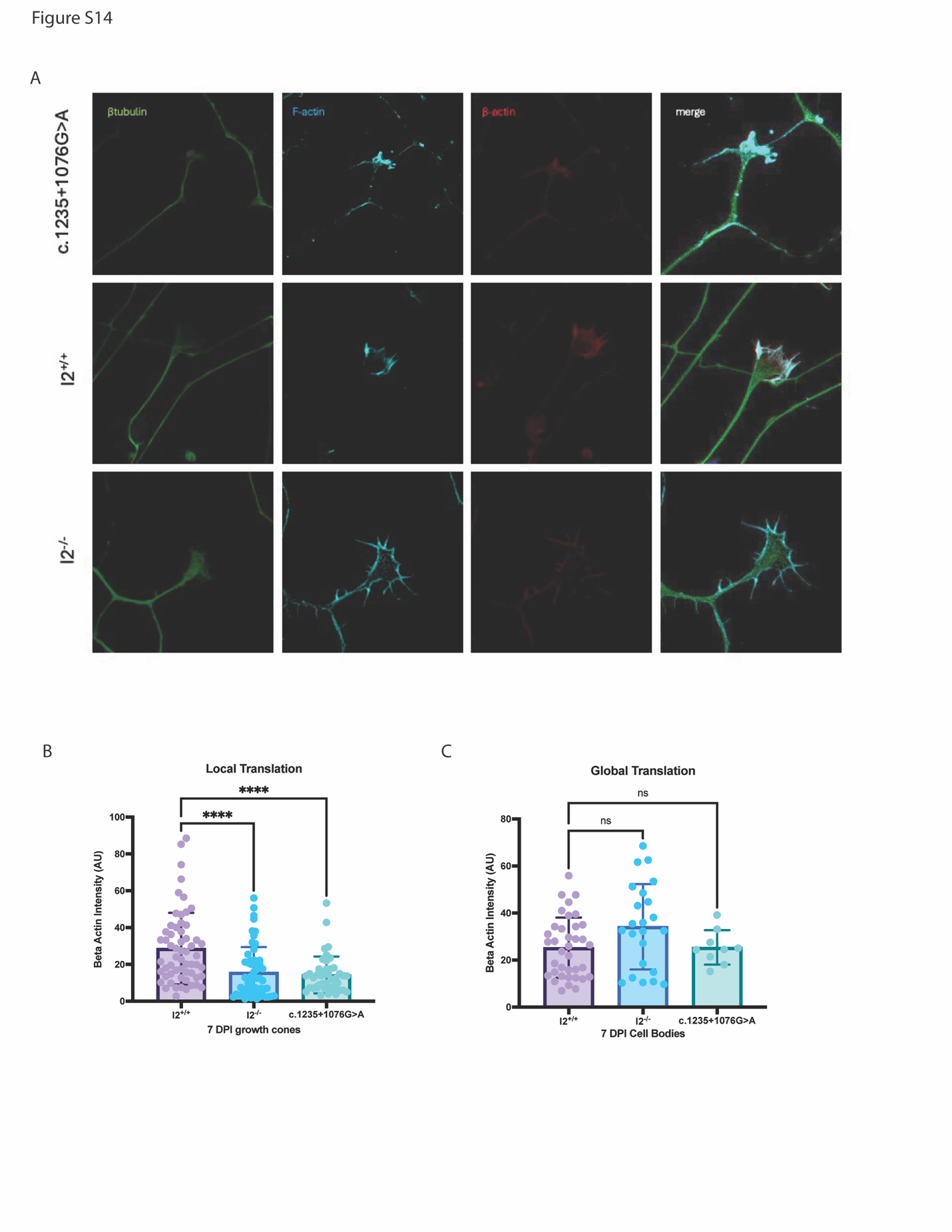

Supplement: Supplement 2 — Figure S1: Validation of iPSC derived iMNs. A) karyotyping of iPSCs for c.1235+450G>A, c.1235+894C>A and c.1235+1076G>A lines revealed no genomic rearrangements. B) iMNs stain positive for motor neuron markers HB9 (red), β-tubulin (green) at five days post differentiation. Figure S2: Presence of biallelic variants in iGHMBP2 allow for phasing of long reads by haplotype. A) long reads for both maternal and paternal alleles for c.1235+450G>A, B) for c.1235+894C>A and C) for c.1235+1076G>A lines. Figure S3: Initial testing of I2-PMO on c.1235+1076G>A fibroblast lines show dose response reduction in IGHMBP2 pseudoexon bands. A) RT-PCR demonstrating reductions of pseudoexon containing band with 200nM-20uM dosing of I2-PMO treatment. STD-PMO (standard control) does not show reduction in pseudoexon band. B) normalization of pseudoexon or wildtype IGHMBP2 bands to GAPDH and to untreated conditions demonstrates statistically significant increase in wildtype band with I2-PMO treatment at concentrations of 200nM and greater (two-way ANOVA with Tukey’s multiple comparisons test, df = 34, p < 0.0001, n=3 replicates per condition). Figure S4: I2-vPMO treatment in patient derived iMNs does not raise total IGHMBP2 mRNA levels. Normalized IGHMBP2 counts do not increase significantly with I2-vPMO treatment compared to STD-vPMO or NT conditions for any cell line. Figure S5: Long read RNA-seq of successfully treated variants complement short-read RNA-seq. A) I2-vPMO treatment in c.1235+1076G>A iMNs increases canonical splicing of exon 8 to exon 9, and no intronic usage is observed. B) A SNP present in exon 13 (black arrow, green SNP) phases with the c.1235+1076G>A allele. In I2-vPMO treated cells, the two reads with canonical splicing come from the intron 8 variant allele. In the STD-vPMO and NT conditions, reads phasing with the c.1235+1076G>A variant are not present or present at low levels, suggesting efficient degradation of pseudoexon containing isoforms. The reads with intronic e [file media-2.docx]
